# Theoretical Evaluation of Polyelectrolyte Layering during Layer-by-Layer Coating of Ultrafiltration Hollow Fiber Membranes

**DOI:** 10.3390/membranes11020106

**Published:** 2021-02-02

**Authors:** Jakob Stumme, Omjothi Ashokkumar, Saskia Dillmann, Robert Niestroj-Pahl, Mathias Ernst

**Affiliations:** 1 DVGW Research Centre TUHH, Am Schwarzenberg-Campus 3, 21073 Hamburg, Germany; mathias.ernst@tuhh.de; 2 Institute of Water Resources and Water Supply, Hamburg University of Technology, Am Schwarzenberg-Campus 3, 21073 Hamburg, Germany; omjothi.ashokkumar@tuhh.de (O.A.); saskia.dillmann@tuhh.de (S.D.); 3 Surflay Nanotec GmbH, Max-Planck-Str. 3, 12489 Berlin, Germany; R.Niestroj-Pahl@surflay.com

**Keywords:** layer-by-layer assembly, polyelectrolyte multilayers, membrane modification

## Abstract

Layer-by-layer (LbL) modification of porous membranes for water filtration has become an active research field in the past few years. Different mechanisms regarding polyelectrolyte film growth, swelling and smoothing, transport through these films, etc., have been studied. Although there are conjectures, it is not yet fully understood where the polyelectrolyte layering takes place when modifying porous membranes, either within the pores or on top of the porous material. This study presents a theoretical approach to investigate the dominant layer buildup regime between pore-dominated vs. layer-dominated growth of polyelectrolytes on porous membranes without mechanically interfering or damaging the membrane material. For this, fouling mechanism processes are used as an analogy. The presented approach gives a new insight into layering conformation and might be helpful to investigate the interaction between the membrane surface and the PE film. Moreover, the MgSO_4_ rejection behavior of two types of modified membranes was investigated: one with an initial pore-dominated layer growth followed by a layer-dominated film growth; the other one with a completely layer-dominated film growth. The data confirm that a rejection for MgSO_4_ could only be achieved in the regime of layer-dominated film growth. Additionally, when layer-dominated film growth prevails from the early stages of the coating process, permeability values are higher at similar MgSO_4_ rejection rates compared to an initial pore-dominated and then layer-dominated film growth. Accordingly, the interaction between the membrane pore size and molecular weight of the polyelectrolytes in the coating solutions plays an important role during LbL coating.

## 1. Introduction

Increasing concentrations of dissolved water substances in raw waters for drinking water production pose an upcoming challenge for water suppliers. Some of these water constituents (e.g., dissolved organics, sulfate, nitrate) cannot be removed by conventional treatments such as media filtration. Therefore, different, more advanced treatment technologies are needed. With the possibility to remove dissolved compounds, for example, divalent ions such as sulfate (SO_4_^2−^), nanofiltration (NF) and low-pressure reversed osmosis (LPRO) are promising and safe alternatives for water treatment [1]. Additionally, NF and LPRO membrane processes find applications in recovery of industrial wastewater, treatment of municipal wastewater, or in pharmaceutical and biotechnological application [2]. However, these processes require high specific energy demands and a certain raw water quality. In contrast, porous membranes such as ultrafiltration (UF) or microfiltration (MF) have larger pores and can often be backwashed due to their material and module properties. Backwash bears the opportunity to treat waters with particle loads, for example, raw waters for drinking water, or industrial and municipal wastewater, and may extend the membrane lifetime [3]. However, UF and MF are not suitable to retain dissolved compounds but present a pretreatment for NF and LPRO [4].

### 1.1. Layer-by-Layer Process and Influences

The layer-by-layer (LbL) coating method offers the option to tailor membranes for water treatment with desired filtration characteristics [5]. For example, membranes were tailored successfully by combining the rejection behavior of membranes in the NF range for divalent ions while keeping the possibility of backwashing [6,7,8]. During the LbL process, a membrane with a certain surface charge is exposed to polycation and polyanion solutions alternatingly. Due to electrostatic interaction, charge compensation, and the release of counterions into the bulk solution, the polyelectrolytes (PE) adsorb onto the membrane surface [9,10]. The process is repeated until the desired film characteristics are obtained.

Different research groups studied the impact of different factors during LbL coating; for example, the impact of ionic strength added to the PE solutions while coating the membrane (also called background ionic strength). Increasing the salt concentration may lead to an increase in the polyelectrolyte multilayer (PEM) thickness and roughness and appears to also influence the density of the film [11,12,13,14,15]. These effects studied on membranes have shown that at similar PE mass adsorptions, lowering the ionic strength of the PE solution results in a lower permeability. This effect is attributed to the lower solubility and diffusivity of water due to a denser film structure [12,15,16]. These studies refer to completely coated membrane surfaces. Additionally, the growth behavior of PEM is influenced by the background ionic strength. Some studies claim that increasing the background ionic strength above a certain concentration leads to an initial exponential growth compared to a linear growth observed at low ionic strength [9,14,17]. Besides the concentration, the type of ions present in the coating solution seems to be another important factor influencing the thickness, the deposited mass [18], and the roughness of the PEM [19]. The molecular weight (MW) of the PE in the coating solution might be another factor influencing PEM growth. According to other studies for the PE pair poly(diallyldimethyl-ammonium chloride) (PDADMAC) and poly(sodium 4-styrenesulfonate) (PSS) at a MW below a certain threshold value (80 kDa for PDADMAC and 25 kDa for PSS), the number of double layers changes when a transition from parabolic to linear growth occurs in the coating process. The transition appears earlier when the PDADMAC MW is lower than the threshold value and later when the PSS MW is below the threshold MW. Further, the PEM layer thickness was observed to be different for these MWs [20,21].

Despite several studies and conjectures, it is not yet fully understood where the adsorption of the PE takes place during membrane modification. Harris et al. (2000) observed that during the buildup of PAH/PSS films on porous membranes, although the molecular weight of the PE is in the range of the molecular weight cut-off (MWCO) of the membrane, only a small part of the inner porous structure is coated, while the major buildup happens on top of the membrane’s active material [22]. As the used measurement technique, field emission scanning electron microscopy (FESEM), requires a mechanical interference, the membrane might not be usable after analysis. Therefore, this technique does not allow further use of the investigated membrane. In contrast, Malaisamy et al. (2005) investigated the buildup of poly(acrylic acid) (PAH) and PSS thin films on a PES support without mechanically damaging the membrane substance. They observed that above a certain MWCO of the virgin membrane, the rejection for sulfate decreases while the permeability remains in a similar range. This behavior in permeability along with the rejection was attributed to a layering of PE within instead of above the porous structure, suggesting the permeability and rejection rate as indications of the layering location [23]. Additionally, membranes were modified using the dynamic layering process, where PE are deposited under applied pressure of the coating solution. These membranes appear to be more stable against hydraulic backwashing and show higher rejections for divalent ions compared to membranes coated without applied pressure. These effects are attributed to the PE layering within the porous structure enhancing the interaction between the membrane’s material and the PEM [24]. Besides the interaction between PE with each other, the interaction between PE on the membrane’s surface and their adsorption location obviously play a major role in the performance of LbL-coated membranes.

**Figure 1:**
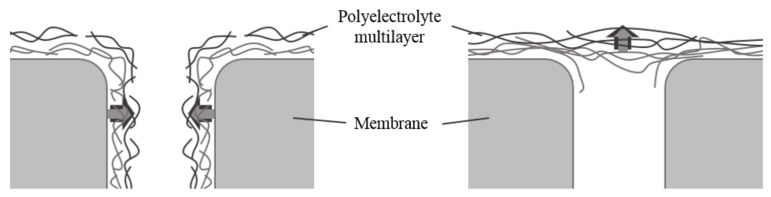
Schematic of pore-dominated (**left**) and layer-dominated (**right**) polyelectrolyte multilayer (PEM) buildup.

De Grooth et al. (2015) and Menne et al. (2016) also focused on the permeability as an indicating parameter for the layering location using the so-called odd-even effect. This effect describes a zig zag pattern for the permeability when coating single PDADMAC/PSS PE layers on top of a membrane surface [6,12], which is attributed to the outermost PE layer of the LbL coating. This layer plays a major role on film growth, water mobility, density, and PEM thickness [12,15,16,25,26,27]. De Grooth et al. (2015) and Menne et al. (2016) coated membranes with PE in low-ionic strength solutions. They observed at low layer numbers that permeability increases when adding an additional PSS layer on an existing PDADMAC/PSS film. This observation is attributed to the PE layering within the porous structure. As the film is claimed to be thinner with PSS as the outermost PE layer, the pore diameter becomes larger compared to PDADMAC as the outermost layer, even though another layer is added. When coating at a higher ionic background strength, which leads to an overall thicker PEM structure, the odd-even effect reverses after a certain number of layers; permeability decreases with an additional layer of PSS. This reversal of the odd-even effect is attributed to a PEM buildup above the porous structure instead of within, as the film structure of the PEM on top of the membrane surface becomes denser [6,12]. To describe the different locations of PEM growth, de Grooth et al. (2015) introduced the expressions of pore- vs. layer-dominated growth regimes for LbL-coated membranes (Figure 1). While the former refers to whether the PE adsorbs onto the inner surface of the membrane, successively decreasing the membrane pore diameter (pore-dominated), the latter refers to PE adsorption on top of the membrane surface, building up a film layer (layer-dominated) [12].

### 1.2. Membrane Fouling and Modeling

During membrane filtration processes of natural water, fouling occurs due to accumulation of substances on the membrane surface. These substances are either sterically rejected or adsorbed on the membrane material due to physical or physiochemical interaction [28]. Internal fouling expresses deposits of foulants within the porous structure of the membrane. External fouling refers to the fouling that occurs on top of the active membrane surface [29].

Multiple studies have investigated and described the fouling processes mathematically. Throughout these studies, four main blocking laws were established to describe the fouling of porous membranes during filtration: (i) complete pore blocking, (ii) standard pore blocking, (iii) intermediate pore blocking, and (iv) cake filtration [30]. The standard pore blocking model assumes that the foulants accumulate within the porous structure, reducing the pore diameter evenly. The complete pore blocking model describes that during fouling, substances adsorb onto the membrane surface, completely blocking available pores. The intermediate pore model describes a similar layering of foulants onto the membrane, while clogging a pore completely. In contrast to the complete pore blocking model, it assumes that foulants deposit also on top of other foulants and do not necessarily block open pores. The cake filtration model suggests that a uniform cake layer is formed on top of the membrane active surface throughout the whole membrane filtration area [29]. Lee et al. (2008) further investigated three of these four models, namely, standard pore blocking, complete pore blocking, and cake layer formation, to describe the fouling of submerged microfiltration hollow fiber membranes [31]. Davis (1992) described several models for the cake layer formation of microfiltration membranes. One of these addresses the cake layer buildup on capillary membranes [32]. The present work assumes that PE multilayer films are particle-free and show a more gel-like film structure. Consequently, we neglect models where particles block the pores of the membranes completely. Therefore, only the standard blocking model and the cake formation model are considered and further evaluated. The resulting model adapts to the standard blocking law of Lee et al. (2008) [31] and the cake layer buildup of Davis (1992) [32].

Generally, for both models, the permeate flux at a specific location and a specific filtration time of the membrane process $J\left(x,t\right)$ is assumed to be proportional to the transmembrane pressure $\Delta p\left(x,t\right)$ and the hydraulic resistance $R_{t}\left(x,t\right)$ of the process according to Darcy’s law [32]:

(1) $$J\left(x,t\right)=\frac{\Delta p\left(x,t\right)}{\eta R_{t}\left(x,t\right)}$$

η: dynamic viscosity.

### 1.3. Standard Blocking Model

If the foulants are small enough to adsorb onto the pore walls, the available pore diameter is reduced. This reduction is related to the mass adsorbed on the pore walls, thus leading to a reduction in the effective pore size and membrane permeability. With the assumptions of a uniform pore size distribution, cylindrical shape, and universal fouling rate for all pores, Lee et al. (2008) described the mass deposited ($m_{d}$) over time related to the pore radius ($r_{p}$) as [31]

(2) $$-\frac{1}{\rho_{d}}\;\;\frac{\delta m_{d}}{\delta t}=N^{\prime }\left(2\pi r_{p}\right)l\frac{\delta r_{p}}{\delta t}$$

*N′*: number of pores, l: pore length, $\rho_{d}$: density of deposits.

With the standard blocking constant $K_{s}=\frac{A_{m}}{\pi N^{\prime }l\rho_{d}r_{p,0}^{2}}$ and the active membrane surface ($A_{m}$), this leads to the reduction in pore diameter with the mass deposited on the membrane surface as [31]

(3) $$\frac{r_{p}\left(x,t\right)}{r_{p,0}}\;={\left(1-\frac{K_{s}m_{d}\left(x,t\right)}{A_{m}}\right)}^{\frac{1}{2}}$$

Using the Hagen–Poiseuille equation for flow through the pores, the permeate flux *J*(*x*, *t*) results as [31]

(4) $$J\left(x,t\right)=\frac{1}{A_{m}}\frac{\pi \;r_{p}^{4}\left(x,t\right)\;N^{\prime }}{8\eta l}\;\Delta p$$

### 1.4. Cake Layer Formation

For the cake layer formation, it is assumed that substances depositing onto the membrane surface build up a uniform cake layer. This cake layer causes an additional local hydraulic resistance ($R_{c}$) to the present membrane resistance ($R_{m}$). For flat membranes, the cake resistance is proportional to the cake thickness or cake mass deposited. For capillary membranes, the overall cake resistance is not only dependent on the film thickness, but also on the decrease in the active surface area due to the curvature. The resistance due to the formation of a cake layer on a cylindrical form is according to Davis (1992) [32]:

(5) $$R_{c}=\rho_{d}\left(1-\epsilon_{d}\right)R_{c}^{\prime }d_{c}ln\left(\frac{d_{c}}{d_{c}-\delta_{c}}\right)={\hat{R}}_{c}d_{c}ln\left(\frac{d_{c}}{d_{c}-\delta_{c}}\right)$$

*𝜖_d_*: void fraction of the cake; $R_{c}$′: specific cake resistance per unit mass per area; $d_{c}:$ capillary diameter; $\delta_{c}$: thickness of the cake layer; ${\hat{R}}_{c}$: specific cake resistance per unit depth.

The overall membrane resistance at a certain time and location ($R_{t}\left(x,t\right)$) is given by [31]:

(6) $$R_{t}\left(x,t\right)=R_{s,n}+R_{c}\left(x,t\right)$$

## 2. Materials and Methods

### 2.1. Materials

Poly(diallyldimethyl-ammonium chloride) (PDADMAC; MW < 100,000 g/mol, 20 wt % solution; MW = 400,000–500,000 g/mol, 20 wt % solution, MW = 40 kDa, 20 wt % solution) and poly(sodium 4-styrenesulfonate) (PSS, MW = 70,000–80,000 g/mol, powder; MW = 1,000,000 g/mol, powder) were purchased from Sigma Aldrich (Taufkirchen, Germany). PSS-Rhodamin (PSS-Rho, MW = 70 kDa) was synthesized by Surflay Nanotec GmbH (Berlin, Germany). Sodium chloride (NaCl; MW = 56,488 g/mol, powder) and magnesium sulfate (MgSO_4_∙7H_2_O; MW = 246.48 g/mol, powder) were purchased from Carl Roth (Karlsruhe, Germany). All solutions were prepared in deionized water (Milli-Q, Millipore Corporation, Billerica, MA, USA). Silica particles with two different pore diameters (7 and 30 nm) were purchased from Silicyle Inc. (Quebec, QC, Canada).

### 2.2. Membrane

Multibore^®^ Polyethersulfone (PES) hollow fiber UF membranes (DuPont/Inge GmbH, Greifenberg, Germany) with two different pore sizes/MWCOs were used (Table 1, Figure S1, Supplementary Material). The membranes with seven capillaries (0.9 mm inner diameter) in one fiber, were operated inside-out. To prevent axial leakage, the membranes are potted into PVC connections. For this, the manufacturer uses a special potting resin, which allows a permeation of the resin into the support structure but does not clog the capillaries. The overall length of the membrane fiber was 30 cm, leading to a total surface area of approximately 60 cm^2^. Data on the capillary diameter, the MWCO, and the porosity are according to the manufacturer’s information. The resulting pore diameter was derived from the MWCO. Porosity was fitted to initial flux values. The thickness of the membrane active separation layer was derived from scanning electron microscopy (SEM) pictures of the 100 kDa membrane. Membranes were rinsed by filtration with deionized water before usage to remove all residues on the surface and in the porous structure.

### 2.3. Modification

**Figure 2:**
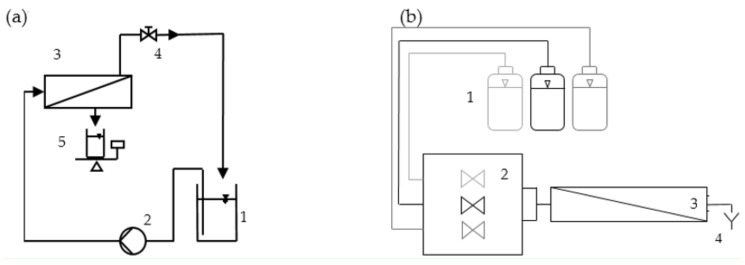
Schematic figure of the (**a**) filtration setup (1: feed tank; 2: feed pump; 3: membrane module; 4: retentate valve; 5: permeate tank) and (**b**) coating setup (1: pressurized solution containers; 2: nanocoater; 3: membrane module; 4: waste disposal).

The membranes were coated using the Surflay Nanocoater (Surflay Nanotec GmbH, Berlin, Germany; Figure 2b). First, the capillaries of the membrane fiber were filled with PDADMAC solution (1 g/L in 0.1 M NaCl). After a contact time of 5 min for membranes with an MWCO of 220 kDa and two minutes for membranes with an MWCO of 100 kDa, the capillaries were rinsed and filled again with fresh PE solution. Conducting the rinsing with fresh solution assured that no potential adsorption sites of the membrane were missed. After another 5 min contact time, the capillaries were rinsed with DI water to remove excess PE solution. The whole procedure was repeated analogously with PSS solution (1 g/L in 0.1 M NaCl). One cycle for PDADMAC and PSS together is referred to as one double layer. After the modification step, DI water permeability was tested, and the coating was repeated accordingly.

### 2.4. Filtration Setup

The DI water permeability was measured in a lab-scale filtration setup (Figure 2a) using deionized water. The solution was pumped into the capillaries of the membrane. One part of the feed was recirculated through a retentate valve back into the feed tank. Permeate was filtered through the membrane and measured gravimetrically. The transmembrane pressure (TMP) was maintained throughout the filtration process. When possible, the TMP was set to approximately 2.5 bar. Due to the high permeabilities at the low coating numbers, a TMP of 2.5 bar was not always achievable. In this case, the pressure was reduced, so membranes were always filtrated in a cross-flow condition (u = 0.25 m/s). For comparison, all flux data were normalized to a TMP of 1 bar to achieve DI water permeability data.

For sulfate rejection measurements, a solution containing 100 mg/L SO_4_^2−^ from MgSO_4_ was used. A volume of 400 mL was used as feed from which a volume of 100 mL permeate was collected, simulating a water conversion of 25%. Similar to DI water filtration, the filtration was carried out preferably at a constant pressure of 2.5 bar. Further, in these experiments, the pressure of 2.5 bar could not always be maintained, due to high flux rates. The rejection rate ($R=1-\frac{c_{p}}{c_{f}}$) was calculated based on conductivity values for the feed and permeate.

### 2.5. Fluorescence Measurement 

To further visualize whether the assimilation of PE is on top or within porous structures, fluorescence measurements were conducted. Porous silica particles with two different pore diameters were coated with fluorescent PSS. According to the equation from Crittenden et al. (2012) ($d_{p}=0.11\cdot Mw^{0.46}$ [33]), both, particles with a smaller pore diameter ($d_{p}$ = 7 nm; equivalent MW ~ 8.3 kDa) than the coating PE MW and particles with a much bigger pore diameter ($d_{p}$ = 30 nm; equivalent MW ~ 200 kDa) than the PE MW were used. The particles with a defined pore size were first rinsed with DI water to remove all possible residuals. As the silica particles show a negative zeta potential at pH 7, the particles were first immersed in 0.1 g/L PDADMAC (MW = 40 kDa) in 0.1 M NaCl for ten minutes. After three times of rinsing with DI water, the particles were then immersed in 0.1 g/L PSS-Rho (MW = 70 kDa) in 0.1 M NaCl. The particles were rinsed again with DI water to remove excess PE from the surface. Particles were then cut and analyzed with a confocal laser scanning microscope (Leica, Wetzlar, Germany) at an extinction wavelength of 530 nm for rhodamine.

## 3. Development of the Layering Model

During the LbL coating process, the PE adsorbs onto the membrane material. This process can be compared to adsorptive fouling during membrane filtration. To describe the layering of PE during LbL coatings, the previously described equations for the standard blocking model from Lee et al. (2008) (within the pores) and cake filtration from Davis (1992) (above the porous structure) are adapted [31,32]. The behavior of the flux and the hydraulic resistance could be evaluated by means of a layering pattern analogous to both fouling models and by regarding the adsorption location. It is assumed that the pores are cylindrical and the PE layer formed is constant in thickness. It is also assumed that no pores are completely blocked and thus dead for filtration during LbL coating. This means that the PE layer deposition either takes place within the porous structure, reducing the pore diameter, or on top of the active layer, forming a uniform cake layer. Due to the combination of both models, both processes can occur at the same time. We also assume an equal wetting of the membrane surface with the PE solution and a uniform charge distribution. This means that the layering occurs similarly throughout the whole membrane material; therefore, the factor of the tangential location along the membrane can be neglected.

Additionally, a new introduced share factor, which is explained later, can visualize where the PEM buildup takes place. It shows the transition from pore- to layer-dominated buildup of the PE film, giving insight into the layering location. 

The standard blocking model is dependent on the mass deposited on the membrane and the pore blocking constant $K_{s}$. As $K_{s}$ in Equation (3) is not changing throughout time, the only factor influencing the decrease in pore diameter is the mass deposited. As the reduction in pore radius is happening during the LbL coating procedure, the radius of the pores decreases with the double layer number n.

(7) $$\frac{r_{p,n}}{r_{p,0}}={\left(1-\frac{K_{s}m_{d}n}{A_{m}}\right)}^{\frac{1}{2}}={\left(1-\frac{{\hat{K}}_{s}n}{A_{m}}\right)}^{\frac{1}{2}}$$

${\hat{K}}_{s}$: standard blocking constant per unit mass.

The resulting flux is calculated analogous to Equation (4) and the resulting hydraulic resistance $R_{s,n}$ is calculated according to Equation (1).

For the cake layer formation, the additional hydraulic resistance is mainly dependent on the layer thickness $\delta_{c}$. As the layer thickness per double layer of PE during formation is assumed to be constant, the specific cake layer resistance after each double layer is calculated according to Equation (5):

(8) $$R_{c,n}={\hat{R}}_{c}d_{c}\ln\left(\frac{d_{c}}{d_{c}-{\delta^{\prime }}_{c}\cdot n}\right)$$

*𝛿_c_^′^*: specific double layer thickness; ${\hat{R}}_{c}\left(=\rho_{d}\left(1-\epsilon_{d}\right)R_{c}^{’}\right)$: specific cake resistance per unit depth.

The total resistance $\left(R_{t}\right)$ of the membrane including the cake layer formed on top of the membrane is according to Equation (6), where $R_{s,n}$ is assumed to be the resistance of the membrane when the transformation from standard blocking to cake layer formation takes place.

(9) $$R_{t}=R_{s,n}+R_{c,n}$$

As these equations each only focus on one of the deposition phenomena, we introduce a new factor $f_{s}$ to combine both processes. The factor $f_{s}$ expresses the share of the standard blocking model on the overall flow through the membrane. Therefore, it describes the shift from the standard to the cake layer blocking model according to its value. The course of this factor is therefore crucial for the determination of the layering location. When the value is close to one, a pore-dominated layer buildup prevails as PE formation is analogous to the standard blocking model. Values close to zero indicate a layer-dominated PEM formation as the equation analogous to the cake layer buildup describes the resulting flux behavior of the membrane. The share factor is calculated according to

(10) $$f_{s}=\frac{1}{1+e^{s_{b}\cdot n-s_{a}}}$$

$s_{b}$: transition sharpness; $s_{a}$: transition factor.

Therefore, the overall membrane resistance at a certain number of coated double layers is calculated as

(11) $$R_{m,n}=f_{s}\cdot R_{s,n}+\left(1-f_{s}\right)\cdot R_{c,n}$$

The corresponding flux is calculated according to Equation (1).

**Figure 3:**
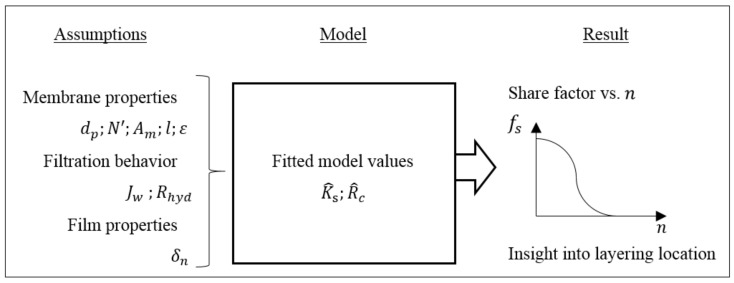
Ingoing assumptions for the model, fitted values during modeling, and result of the model.

Overall, with initial assumptions, and fitted values according to experimental data, the model visualizes the predominant layering location, expressed by the share factor $f_{s}$ (Figure 3).

## 4. Results and Discussion

Results are assigned to the following designation: M/E_220/100–PSS/PDADMAC (model/experimental results_MWCO of the virgin membrane–PE of the outermost layer).

The model fitting was performed so that a maximal correlation coefficient $R^{2}$ was reached regarding both data, the membrane resistance and membrane permeability. The correlation coefficient for the membrane resistance is calculated as follows:

(12) $$R^{2}=\frac{\Sigma \left(R_{E}-{\bar{R}}_{E}\right)\left(R_{M}-{\bar{R}}_{M}\right)}{\sqrt{\Sigma {\left(R_{E}-{\bar{R}}_{E}\right)}^{2}\Sigma {\left(R_{M}-{\bar{R}}_{M}\right)}^{2}}}$$

*R_E_*: experimental resistance data; $R_{M}$: model resistance data; ${\bar{R}}_{i}$: mean values

The normalized flux values are calculated analogously.

Additionally, a chi-squared value (${Χ}^{2}$, Equation (9)) is calculated due to the non-linear form of the model. According to Usman et al. (2018), it indicates the bias between model and experimental results. Therefore, in addition to $R^{2}$, the ${Χ}^{2}$ value was also minimized for both the normalized flux and the membrane resistance [34].

(13) $${Χ}^{2}=\Sigma {\left(\frac{R_{E}-R_{M}}{R_{M}}\right)}^{2}$$

### 4.1. Normalized Flux, Hydraulic Resistance, and Share Factor

**Figure 4:**
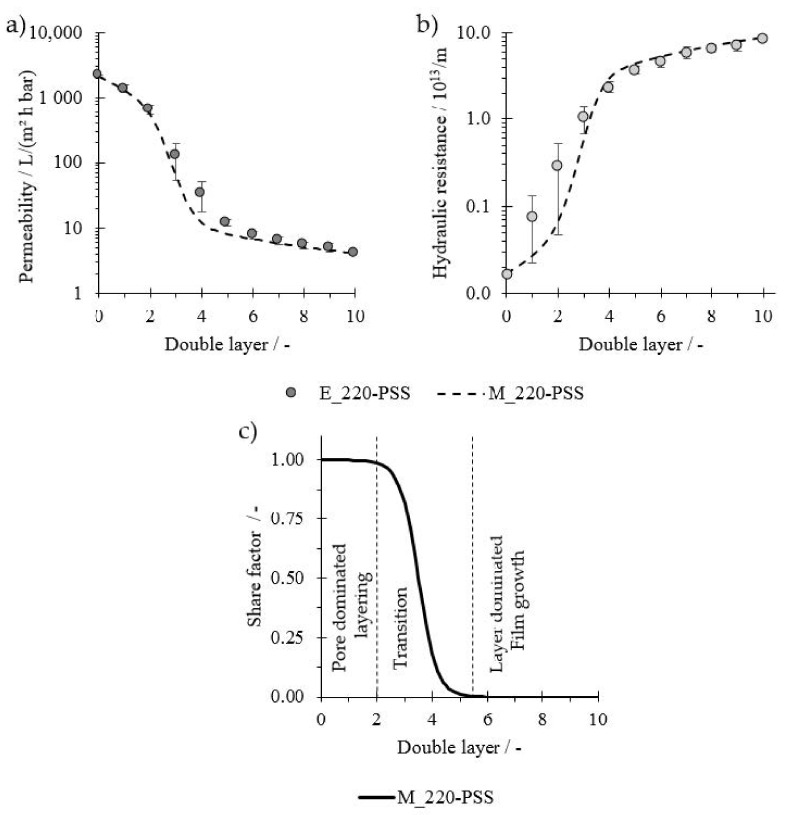
(**a**) Permeability and (**b**) hydraulic resistance for the coating of a membrane with a nominative molecular weight cut-off (MWCO) of 220 kDa coated with polyelectrolytes (PE) with high molecular weight (MW); (**c**) share factor fs of the model indicating the share of the standard blocking model and therefore the share of pore-dominated layer buildup; n = 2.

First, the described model (M_220-PSS) is compared to experimental values of membranes with a 220 kDa nominative MWCO coated with PE of low molecular weight (PDADAMA < 100 kDa, PSS 80 kDa) and PSS as the outermost layer (E_220-PSS). The membrane properties are displayed in Table 1. Further required assumptions are listed in Table 2 together with the fitted model parameters. Flux data, membrane resistance, and the respective model values are displayed in Figure 4a,b, respectively. The experimental data show a good reproduction as displayed by the small error bars. Both experiments show drastic flux decreases with each double layer in the early stages of the coating process, indicating the successful deposition of PE on the membrane. After a certain number of double layers, the gradient of the flux decline becomes less pronounced. The increase in membrane resistance appears to be rather exponential for the early stages of the coating, although initial values are comparably small. The increase in hydraulic resistance then shifts to a rather linear course as the coating proceeds. With the right fitting parameters, the model (line) is in good agreement with the experimental data (markers).

The share factor $f_{s}$ is displayed in Figure 4c. For the experimental data, the standard blocking model is the dominating model to describe the decrease in flux with each double layer until the second, as $f_{s}$ is close to one. Therefore, it indicates that the PE mainly adsorb within the membrane structure, indicating a pore-dominated layer buildup of the PE film in the early stages of the coating. 

The decrease in $f_{s}$ between the second and fifth double layers indicates the shift from standard blocking to cake layer formation. From the fifth double layer onwards, as $f_{s}$ is close to zero, the change in resistance and flux is best described by the cake formation, which indicates that the PE form a dense layer on top of the membrane and therefore a layer-dominated film buildup is present. This shift after a certain double layer number agrees with expectations. The MW of PE is lower than the MWCO of the membrane. Therefore, it is evident that PE would deposit within the porous structure as they are not large enough to cover the pores completely. With this, the pore diameter is successively reduced, so that at some point the pores are so small that PE accumulate on top. According to the model, this would happen at a pore radius of 10–12 nm. This is according to the equation from Crittenden et al. (2012) ($d_{p}=0.11\cdot Mw^{0.46}$) [33] in the MW range of approximately 80–120 kDa. As this is the range of the used MW of the PE, the shift appears to be plausible. To investigate if the model can be applied when relations of the MWCO and PE MW are vice versa, membranes with an MWCO of approximately 100 kDa were coated with PE with a higher MW (MW_PSS_ = 1000 kDa; MW_PDADMAC_ = 400–500 kDa). In addition, these membranes were tested after each single layer to investigate the odd-even effect on permeability. Membrane properties are listed in Table 1. Further assumptions and resulting fitted values are listed in Table 2.

**Figure 5:**
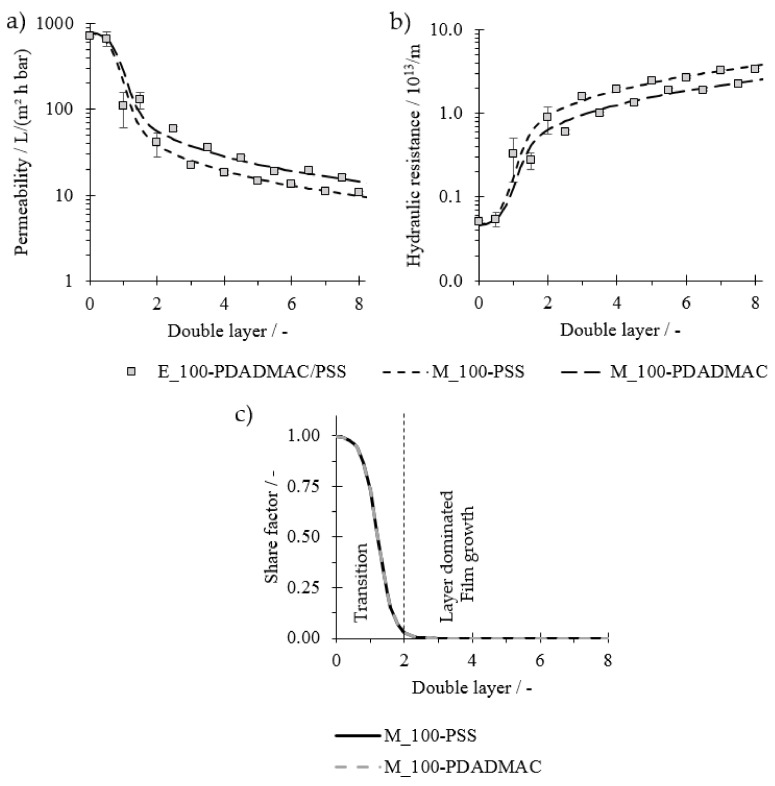
(**a**) Permeability and (**b**) hydraulic resistance for the coating of a membrane with a nominative MWCO of 220 kDa coated with PE with high molecular weight; (**c**) share factor fs of the model indicating the share of the standard blocking model and therefore the share of pore-dominating layer buildup; n = 2.

Permeabilities and the hydraulic resistance of the experimental values are displayed in Figure 5a,b: half numbers stand for PDADMAC and whole numbers stand for PSS as it is the outermost layer (E_100-PSS/PDADMAC). In these experiments, there is a sharp decrease in flux during the first few coatings (Figure 5a). The decline in permeability is even more drastic than observed in experiments with E_220-PSS (Figure 4a). The odd-even effect is clearly visible as results show a zig-zag pattern for the development of flux and hydraulic resistance vs. the number of layers. The flux with PDADMAC is generally higher in the same range of double layers compared to PSS as the outermost layer. The hydraulic resistance is accordingly lower with PDADMAC as the final layer. This is in agreement with previous studies when the coating was assumed on top of the porous structure [6,12].

To be able to describe both situations, models were examined separately for the course of PDADMAC (M_100-PDADMAC) and PSS (M_100-PSS) as the determining layer. With the fitted values from Table 2, both models can describe the respective experimental data (Figure 5). The odd-even effect is, in addition to flux and resistance values, also expressed in the values of ${\hat{K}}_{s}$ and ${\hat{R}}_{c}$. From Equations (7) and (8), it can be seen that the layer density is the only parameter influencing both processes, deposition within and above the pores. This leads to the assumption that films with PDADMAC as the outermost layer are less dense (lower ${\hat{K}}_{s}$ and ${\hat{R}}_{c}$), supporting the conclusions from previous studies [12,16].

Figure 5c shows the share factor $f_{s}$ for both cases, PSS (full line) and PDADMAC (dotted line). The share factors for both models decrease sharply from the beginning, indicating that a deposition of PE within the pores is, if at all, only happening in the early stages of the coating. Already after two double layers, the cake formation model is dominating completely, which indicates that deposition of PE occurs overall on top of the porous substrate. This is expected as the PE molecular weight is larger than the MWCO of the membrane. The initial pattern of standard blocking could be due to an adsorption of parts of the PE molecular chain within the porous structure. 

Both graphs of the respective share factors overlap throughout the whole regarded double layer range. This is expected as the same layer buildup is displayed and therefore the same layering location must result.

Overall, these results show that the model can display the shift from the standard blocking to cake formation regardless of the outermost layer and therefore, enables the insight into the layering location.

### 4.2. Plausibility of the Model

The applied models used as the foundation of the developed model were initially derived by investigating the flux of pressure-driven experiments. As both models attribute the flux and resistance behavior to the additional foulant substance accumulating within the structure or on the surface of the membrane, the analogy between LbL PEM formation and fouling deposition is given, even though the conducted LbL coating is a non-pressure-driven process. One double layer therefore compares to one temporary moment during the analogous fouling process of pressure-driven membrane filtration.

The assumed layer thickness of 4 nm per bilayer for the cake layer buildup is in agreement with the literature [9,13,14], though there are contrary statements on the linearity of the overall film thickness increase. Guzmán et al. (2009) observed that up to a concentration of approximately 0.3 M NaCl, the multilayer growth appears to be linear in film thickness with increasing layer number. Exceeding this background ionic strength leads to a shift to a rather exponential growth [9,14]. Tang et al. (2016), on the contrary, observed a rather parabolic growth, implying a growth regime between linear and exponential growth, already at 0.1 M NaCl [17]. Additionally, the first double layers (DL) also have an impact on the growth pattern of PEM. However, at concentrations of 0.1 M NaCl, the influence of the virgin membrane material is stated to be low [13]. Furthermore, PDADMAC as the first layer was observed to lead to a linear growth of the PEM [35]. Overall, even if the layer buildup with a background ionic strength of 0.1 M would follow a parabolic tendency, the used assumption of a linear growth would be appropriate, as the increment of the layer thickness increase with the number of double layers would be low up to 10 DL [17]. For an exponential growth, for example, at high ionic strength, the model would have to be adapted to the increment in the thickness per bilayer. 

Next to the impact of the ionic strength of the PE solution, the chemistry and structure of the membrane material play a role in the modification process, as membrane properties might differ. The pore size distribution is one such critical parameter as the shift from pore-dominated to layer-dominated film buildup might happen over several coating steps. Properties such as charge density, zeta potential, hydrophilicity, or porosity might also influence the layering process [5].

Another impact is the coating technique—dynamic vs. static coating influences the layer buildup of the membrane. In this study, the membranes were statically coated as there was no pressure applied to the PE coating solution. Dynamic coating might lead to higher adsorption rates of PE in the pore, as other studies report high rejection rates at lower layer numbers [15]. 

Therefore, in addition to changing the parameters of the coating solution, the model would have to be adapted and fitted accordingly for different membrane materials and process parameters, in order to determine the predominant layering location.

For the initial flux reduction in the standard blocking model, $K_{s}$ is the dominating parameter. Compared to literature data from Lee et al. (2008), our fitted $K_{s}$ values are in the same range. A direct comparison of these values is critical though, because Lee et al. (2008) regarded the fouling process in a time-and flux-dependent process instead of a layer- and adsorption-dependent process [31]. Values for ${\hat{R}}_{c}$ are higher compared to the literature of Davis (1992). This is, however, not surprising, as Davis was observing cakes formed from micro-sized rigid spheres [32]. We can assume that the void volume between these particles is much higher than for dense PEM, thus causing PEM to result with higher specific hydraulic resistance.

So conclusively, the model, as it is now, provides a systematical method to gain insight on the adsorption location during LbL modification of porous membranes. This knowledge can further give a better understanding of the interaction between PE and membrane surfaces. The insight could help, for example, when, on the one hand, the internal structure shall be coated, while retaining a porous membrane, or, on the other hand, only having a PEM on top of the porous structure as the only hydraulic resistance acting as the active separation layer.

### 4.3. Rejection for MgSO_4_

**Figure 6:**
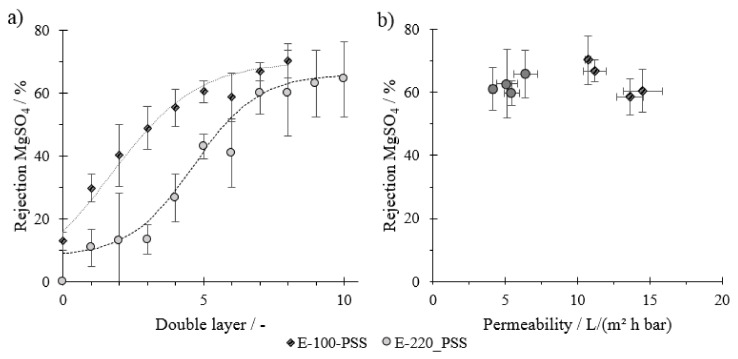
(**a**) Rejection rates for MgSO_4_ with increasing double layer (DL) number and (**b**) MgSO_4_ rejection rates vs. the permeability for the last four DLs for membranes coated with PE smaller than the membrane’s MWCO (Circles) and with PE bigger than the membrane’s MWCO (Squares); n = 2.

Figure 6 shows the rejection values for MgSO_4_ with each full double layer. For both virgin membranes, a rejection for MgSO_4_ of ca. 10% is visible. This could be attributed to electrostatic and charge effects of negatively charged membrane surfaces and SO_4_^2−^. The MgSO_4_ rejection rates for membranes modified with a molecular weight smaller than the membrane’s MWCO remain low with increasing layer number in the range until about three double layers. From the fourth double layer onwards, there is a steep increase in sulfate rejection from ca. 20 to about 60% after seven double layers. After this, the increase in the rejection rate flattens out until the tenth DL.

When modifying the membrane with a PE molecular weight higher than the membrane’s MWCO, a direct increase in rejection for MgSO_4_ can be observed, even with the first DL. The rejection rate then increases nearly linearly until the fifth DL. An increasing rejection tendency with each DL until the eighth DL is observed, but this is less severe with additional layers.

These results additionally indicate that the porous structure of the membrane remains when membranes are coated with a lower molecular weight, as pores are not yet narrow enough to reject divalent ions. In addition, rejection rates start to increase directly when the PE molecular weight is higher than the MWCO, indicating a cake layer formation. Correlating results from the share factor models and rejection rates, we can see that the rejection of divalent ions only starts to occur when the transition from pore-dominated to layer-dominated layering happens. From this point onwards, the membrane could be considered as a dense membrane, where ion transport is based on the solution diffusion processes. With increasing layer thickness, as more double layers are applied, the rejection increases. This is attributed to the diffusive resistance of the material on ion transport. From this, we can further conclude that rejection for divalent ions only takes place when there is an overlay of PE on top of the porous substrate of the membrane. At a certain number of DLs, the rejection stabilizes, which cannot be seen in flux rates and hydraulic resistance values. Both transport mechanisms through a uniform separation layer, hydraulic water flux and ion diffusion, mainly depend on the layer thickness of the film. When both parameters change proportionally with the layer thickness, a certain retention adjusts. This stabilization of the rejection rate (Figure 6) happens at later coating stages than the linearization of the hydraulic resistance (Figure 4b and Figure 5b). A reason for this can be that the flux rate directly influences concentration polarization and with it the permeate quality and diffusion rate of ions. Structural inhomogeneity within the film, for example, as a result of membrane pore size distribution, can be another factor. The zeta potential also has an influence on the separation effect. Several studies on the PDADMAC/PSS system have shown that with an increasing layer number, the zeta potential shifts and influences the rejection behavior [12,15,36,37].

Overall, observed rejections are rather low compared to other studies for the PE system PDADMAC/PSS [6,7,8,12,16]. Reasons for this are likely retention experiments between each coating step. These filtrations in between the modification steps could lead to a more open layer structure and with it a reduced rejection rate. When coated in one consistent series, we could achieve comparable rejection rates (Figure S2, Supplementary Material). An increase in sulfate rejection rates could be achieved adjusting the coating ionic strength, coating under dynamic conditions, or post-modifying the resulting membrane using ion solutions and re-exposure to PSS [12,15,16].

Figure 6b displays rejection rates for the last four DLs of the modified membrane in relation to their permeability. Values show that although rejection rates are similar, the permeability is lower when membranes are coated with a lower PE molecular weight than the MWCO, even though the initial permeability of the virgin material is much higher. This, again, indicates that the PE reduce the pore diameter initially before its buildup on top of the substrate occurs. The support material just before cake buildup has a higher hydraulic resistance, which again results in lower overall membrane permeability. The ratio between the PE molecular weight and membrane MWCO is therefore another critical parameter for the filtration characteristics of the modified membrane.

### 4.4. Fluorescence Measurement

**Figure 7:**
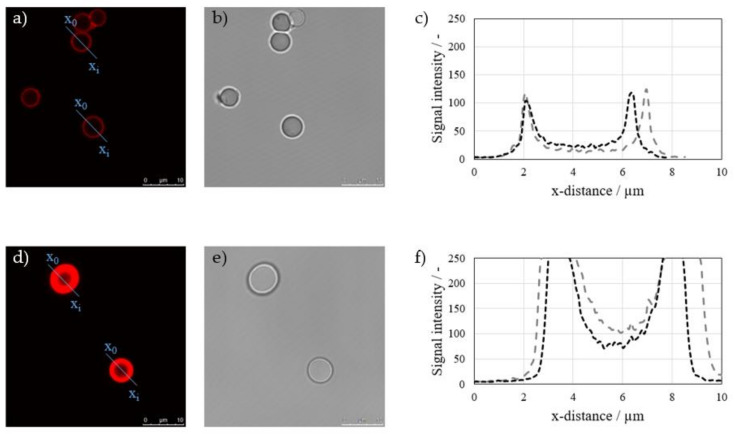
Confocal laser scanning microscope images of cross-sections of coated particles with a pore size smaller than the PE MW (**a**,**b**) and bigger than the PE MW (**d**,**e**); (**c**,**f**) show the detected signal intensity against the distance through the regarded particles.

As PES membranes show an auto-fluorescence in the regarded wavelength of PSS-Rho (MW = 70 kDa), fluorescence measurements were conducted using porous silica particles as a model system. The particles come with a defined pore diameter of 7 nm (equivalent to approximately 8 kDa) and 30 nm (equivalent to approximately 200 kDa). These pore sizes stand exemplary for membranes with either a lower MWCO or membranes with a higher MWCO than the PE MW. After coating, the particles were cut, and cross-sections of the particles were analyzed. Results of fluorescence measurements of different particles with a pore diameter of 7 and 30 nm are displayed in Figure 7a–f, respectively.

Results show that, when coating porous particles with a small pore diameter, the resulting intensity of the fluorescence signal and the visible thickness are low, indicating that a low amount of the PE mass is adsorbed onto the available surface. The narrow peak shapes signify a low penetration depth of the PE into the porous structure of the particles and suggest a layering above or at least close to the particle surface.

Coating porous particles with a larger pore diameter results in a much higher signal intensity, even above detection limits. This indicates that a high amount of PE is adsorbed onto the virgin material. The broad signal intensity peak and the comparatively high intensity even close to the center of these particles further show that PE penetrate deep into the particle structure. As no convectional PE transport takes place during immersion, diffusion is the major mechanism for PE penetration.

Although the wavelength of the confocal laser microscope is much larger than the estimated and expected film thickness, which means that the resolution of this method is too low to give detailed information about the actual quantitative depth of penetration, results can be seen as an indication of the layering location. 

So overall, relating the results for both silica pore sizes to the membrane experiments, they support the interpretation of results. When PE are smaller than the pore diameter, they seem to penetrate into the particle structure, indicating a pore-dominated film buildup, while PE tend to accumulate on top or close to the particle surface when PE are bigger, indicating a layer-dominated film buildup.

## 5. Conclusions

We have introduced a model for the systematic determination of the layering location of PEM on membrane surfaces. For this, fouling analogies to standard pore blocking and cake layer formation models were used. To connect these individual models, we introduced a share factor $f_{s}$. This factor indicates the share of pore reduction on the overall layer buildup of the PEM and can thus visualize the predominant PE layering location: either within or above the membrane pores.

Porous membranes were coated with PDADMAC/PSS with a lower MW than the MWCO of the membrane. The model fits experimental filtration data well. For the first DL, the share factor $f_{s}$ close to one indicates a layering of the PE double layers within the porous structure. Subsequently, after a few DLs, $f_{s}$ shows a sharp decrease close to zero, indicating that the layer formation shifts to a uniform buildup of the PE multilayer above the pores of the membrane. 

Membranes were single layer-coated also with an MW larger than the membrane’s MWCO. The two models could be fitted to both PE, cations and anions, as the outermost layer (odd-even effect). Although fitted values differ for these two models, the course of $f_{s}$ for both models is overlapping throughout the whole double layer range. This means that the model can describe the shift from pore- to layer-dominated PE growth, independent of the outermost PE layer. For both models, $f_{s}$ is instantly shifting from one to zero, designating a direct shift from pore-dominated to layer-dominated PEM growth, indicating that PE with a larger MW than the membrane’s MWCO directly form a PE multilayer above the pores, instead of layering within.

Furthermore, the modified membranes were tested on rejection rates for MgSO_4_. Results support the conclusions from the hydraulic model. If the PE molecular weight is lower than the membrane’s MWCO, there is no initial increase in rejection as pores are still too large. When PE start to accumulate on top of the membrane surface, forming an even layer, MgSO_4_ rejection increases. This increase in rejection is in agreement with the decrease in $f_{s}$ and with the start of layer-dominated PEM growth. Therefore, these results also support the idea that the model can visualize the layering location, expressed by the share factor.

Additionally, at comparable rejection rates, the permeability is lower for modified membranes with the PE MW lower than the membrane’s MWCO, even though the initial permeability of the virgin material is much higher. Therefore, in addition to the results supporting the model, the relationship between the PE MW and the membrane MWCO is identified as another critical parameter for LbL modification of membranes.

Confocal laser scanning microscopy gave a visual indication of the PE layering location on porous particles as a model system. These results once more support data from the filtration experiments and visualization by the share factor. Layering occurs on top of porous substrates when PE are bigger than the pore diameter. When PE are smaller than the pores of the substrate, a deep penetration of PE into the particle structure takes place.

Overall, the introduced model gives the possibility to systematically describe and visualize the shift from a pore-dominated to a layer-dominated film buildup. Therefore, it can give insight into the layering location of PE on porous membranes. With this knowledge, one can specifically modify membranes either to maintain a porous membrane surface and coat the internal membrane structure or build up a dense layer film on top of the membrane from the early stages on. Knowing the layer buildup location may also help to gain further insight into the mechanical stability of PEM on membranes.

## Figures and Tables

**Table 1:** Membrane parameters for two different membranes used in the experiments.

Parameter	Abbreviation	Unit	MB220	MB100
Active layer thickness/pore length	l	µm	2	2
Capillary diameter	$d_{c}$	mm	0.9	0.9
Molecular weight cut-off	MWCO	kDa	220	100
Initial pore diameter	$d_{p,0}$	nm	36	22
Number of pores per unit of active area	$N^{\prime }$	$10^{14}$	2.85	7.63
Porosity	ε	%	29	29

**Table 2:** Assumed and fitted parameters for the models describing the coating process of low-molecular weight (MW) polyelectrolytes (PE) on higher-molecular weight cut-off (MWCO) membranes and a determining layer of poly(sodium 4-styrenesulfonate) (PSS) (M_220-PSS), and the coating process of high-MW PE on lower-MWCO membranes and a determining layer of PSS (M_100-PSS) and poly(diallyldimethyl-ammonium chloride) (PDADMAC) (M_100-PDADMAC).

Assumed Parameters	Unit	M_220-PSS	M_100-PSS	M_100-PDADMAC
Dynamic viscosity (*η*)	$\frac{\mathrm{kg}}{\mathrm{ms}}$	1.002$\cdot 10^{3}$		
Thickness per double layer ($\delta_{n})$	nm	4.0		
Mass deposited per layer (${\dot{m}}_{d})$	kg	0.25		
Fitted parameters				
Standard blocking constant ($K_{s}$)	$\frac{m^{2}}{\mathrm{kg}}$	7.9$\cdot 10^{4}$	4.0$\cdot 10^{4}$	2.5$\cdot 10^{4}$
Specific cake resistance (${\hat{R}}_{c}$)	$\frac{10^{19}}{m^{2}}$	46.2	20.6	7.3
Transition coefficient a ($s_{a}$)		10.5	4.0	4.0
Transition coefficient b ($s_{b}$)		3.0	4.5	4.5
Resulting correlation coefficient and chi-squared value				
Correlation coefficient resistance ($R_{J_{w}}{}^{2}$)		0.999	00.994	0.992
Chi-squared value (${Χ}_{J_{w}}^{2}$)		0.86	0.75	0.62
Correlation coefficient resistance ($R_{R}^{2}$)		0.995	0.994	0.991
Chi-squared value (${Χ}_{R}^{2}$*)*		1.33	0.25	0.37

## Data Availability

The data presented in this study are available on request from the corresponding author.

## Supplementary Materials

The following are available online at https://www.mdpi.com/2077-0375/11/2/106/s1, Figure S1: Scanning electron microscopy (SEM) pictures of the 100 kDa hollow fibre membrane. (a) Crosssection and (b) surface of a dried uncoated membrane, and (c) Surface of a dried, 8 double layer coated membrane (taken from [1]). Figure S2: Rejection rate vs. permeability for experimental data in this study (E-100-PSS, E-220-PSS) and other conducted experiments (Consistent Coating Experiments, n = 5). Membranes of “Consistent Coating Experiments” were modified in one consistent coating process without rejection experiments between each coating step. General coating and filtration parameters were comparable.
