# Supplementary material for: Theoretical Evaluation of Polyelectrolyte Layering during Layer-by-Layer Coating of Ultrafiltration Hollow Fiber Membranes
Source: Membranes (Basel). 2021 Feb 2;11(2):106. doi: 10.3390/membranes11020106 (PMC7913055; doi:10.3390/membranes11020106)
Supplement: Supplementary file 1 [file membranes-11-00106-s001.pdf]

membranes

Supplementary Material: Theoretical Evaluation of Polyelectrolyte Layering during Layer-by-Layer-Coating of Ultrafiltration Hollow Fibre Membranes

Jakob Stumme, Omjothi Ashokkumar, Saskia Dillmann, Robert Niestroj-Pahl and Mathias Ernst

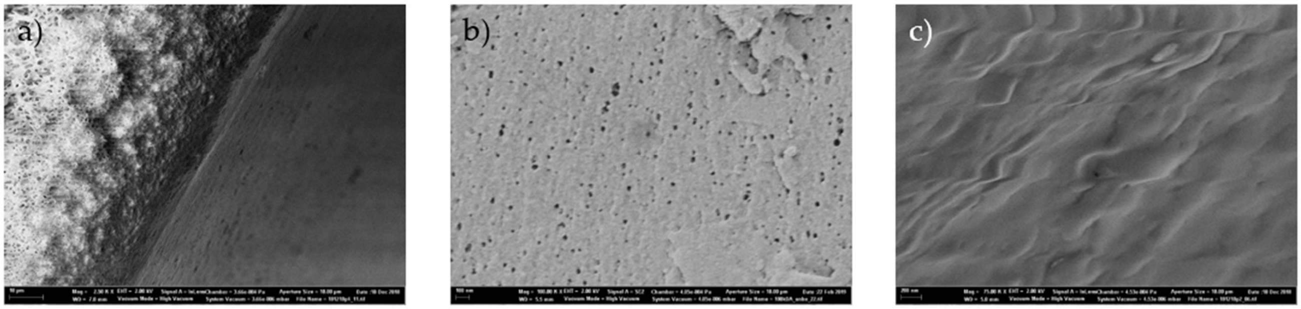

Figure S1. Scanning electron microscopy (SEM) pictures of the 100 kDa hollow fibre membrane. **a**) Crosssection and **b**) surface of a dried uncoated membrane, and **c**) Surface of a dried, 8 double layer coated membrane (taken from [1]).

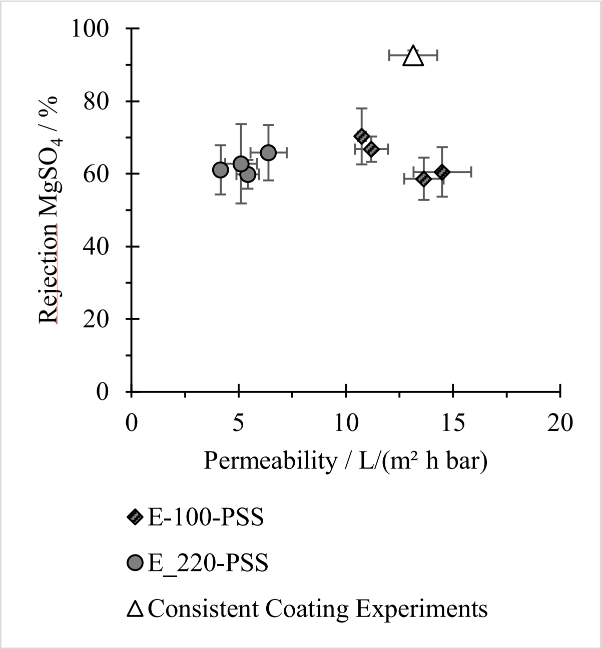

Figure S2. Rejection rate vs. permeability for experimental data in this study (E-100-PSS, E-220-PSS) and other conducted experiments (Consistent Coating Experiments, n = 5). Membranes of "Consistent Coating Experiments" were modified in one consistent coating process without rejection experiments between each coating step. General coating and filtration parameters were comparable.

References

1. Dillmann, S.; Kaushik, S.A.; Stumme, J.; Ernst, M. Characterization and Performance of LbL-Coated Multibore Membranes: Zeta Potential, MWCO, Permeability and Sulfate Rejection. *Membranes* **2020**, *10*, 412, doi:10.3390/membranes10120412.
